# Selection and Verification of Appropriate Reference Genes for Expression Normalization in *Cryptomeria fortunei* under Abiotic Stress and Hormone Treatments

**DOI:** 10.3390/genes12060791

**Published:** 2021-05-21

**Authors:** Yingting Zhang, Lijuan Zhu, Jinyu Xue, Junjie Yang, Hailiang Hu, Jiebing Cui, Jin Xu

**Affiliations:** 1 Key Laboratory of Forest Genetics & Biotechnology of Ministry of Education, Nanjing Forestry University, Nanjing 210037, China; ytzhang@njfu.edu.cn (Y.Z.); zhulijuanlucky@163.com (L.Z.); xjinyu@njfu.edu.cn (J.X.); yangjj@njfu.edu.cn (J.Y.); huhailiang@njfu.edu.cn (H.H.); cuijiebing@163.com (J.C.); 2 Co-Innovation Center for Sustainable Forestry in Southern China, Nanjing Forestry University, Nanjing 210037, China; 3 College of Forestry, Nanjing Forestry University, Nanjing 210037, China

**Keywords:** *Cryptomeria fortunei*, qRT-PCR, reference gene, abiotic stress, hormone treatments, different tissues

## Abstract

*Cryptomeria fortunei* has become one of the main timber afforestation species in subtropical high-altitude areas of China due to its fast growth, good material quality, and strong adaptability, showing broad application prospects. Quantitative real-time PCR (qRT-PCR) is the most accurate and widely used gene expression evaluation technique, and selecting appropriate reference genes (RGs) is essential for normalizing qRT-PCR results. However, suitable RGs for gene expression normalization in *C. fortunei* have not been reported. Here, we tested the expression stability for 12 RGs in *C. fortunei* under various experimental conditions (simulated abiotic stresses (cold, heat, drought, and salinity) and hormone treatments (methyl jasmonate, abscisic acid, salicylic acid, and gibberellin) and in different tissues (stems, tender needles, needles, cones, and seeds) using four algorithms (delta Ct, geNorm, NormFinder, and BestKeeper). Then, geometric mean rankings from these algorithms and the RefFinder program were used to comprehensively evaluate RG stability. The results indicated *CYP*, *actin*, *UBC*, and *18S* as good choices for studying *C. fortunei* gene expression. qRT-PCR analysis of the expression patterns of three target genes (*CAT* and *MAPK1/6*) further verified that the selected RGs were suitable for gene expression normalization. This study provides an important basis for *C. fortunei* gene expression standardization and quantification.

## 1. Introduction

Quantitative real-time PCR (qRT-PCR) has the characteristics of high sensitivity, high efficiency, and convenient operation and can be used to accurately analyze experimental results [1,2,3,4]. Currently, it is one of the most commonly used methods and the most important method for investigating gene expression. However, qRT-PCR results are affected by many variable factors, such as RNA template, reverse transcription efficiency, primer specificity, protocol variability, and data normalization and analysis method. The main problems caused by inconsistent data normalization and analysis are widely ignored [3]. Therefore, it is very important to compare the expression levels of all tested genes with the reference genes (RGs) to maximize the reproducibility of data analysis to obtain more accurate and reliable analysis data.

An increasing number of studies have now shown that the most commonly used RGs in plants, such as the *glyceraldehyde-3-phosphate dehydrogenase* (*GAPDH*), *actin*, *18S ribosomal RNA* (*18S rRNA*), *β-tubulin* (*TUB*), and *transcription elongation factor* (*EF1α*) genes [5,6,7,8], show large differences in applicability [9,10]. For example, the expression patterns of *18S* are unstable during the development of Chinese cabbage (*Brassica rapa*) flower buds [7] and under abiotic stress in moss (*Syntrichia caninervis*) [11] but stable in different tissues and under different treatments in millet (*Panicum miliaceum*) [12]. Perfect RGs are supposed to be expressed stably and constitutively in different tissues, as well as under all physiological conditions; unfortunately, there are no genes that are absolutely stably expressed. More specifically, the so-called stable expression of any RG occurs only in specific tissues or under specific environmental conditions [13]. Therefore, it is necessary to test the expression stability of multiple candidate RGs in different tissues or under different environmental conditions before conducting qRT-PCR experiments. To date, there are many reports on the screening of plant RGs under various conditions, including under (a)biotic stress and during growth and development in different tissues and varieties [11,13,14,15]. For example, Ni et al. [1] studied cotton rose (*Hibiscus hamabo*) under abiotic stress and found that *actin* and *ski-interacting protein (SKIP*) can be considered the best RGs for the analysis of gene expression. However, suitable RGs that can be used for normalization of gene expression in Chinese cedar (*Cryptomeria fortunei*) have not been reported.

*C. fortunei*, belonging to Taxodiaceae, is a species that is endemic to China. It has become one of the main fast-growing timber afforestation species in subtropical high-altitude areas in China because of its fast growth, straight trunk, and good texture, with broad application prospects. *Cryptomeria* prefers a warm and humid climate, and its growth is often affected by adverse environmental conditions, such as low temperature, drought and acid stresses, and hormone treatments [16,17,18]. For example, low temperature (4, 0, −4, −8, −12, −16, and −20 °C) for 2, 12, or 24 h significantly decreased the chlorophyll (chl) content (chl a, chl b, and chl a + b) and chl fluorescence parameters (such as maximum quantum yields of photosystem II (*F*_v_/*F*_m_)), increased electrolyte leakage, and damaged needle chloroplast ultrastructure of *C. fortunei*, affecting its growth [17]. Li et al. treated *C. japonica* with different low temperatures (0, −5, −10, and −15 °C) for 12 h, and also found that the electrolyte leakage and malondialdehyde (MDA) of the needles gradually increased with the decrease of the treatment temperature, while the free proline (Pro) and the content of soluble sugars increased first and then decreased with the decrease of the treatment temperature [19]. It can be seen that plant cell membrane permeability increases, intracellular electrolytes extravasate, and osmotic adjustment substances increase under low temperature, which prevents damage to cell structure and enhances protection. Futamura et al. [20] isolated three thaumatin-like protein (TLP)-encoding cDNAs (*Cryj 3.1*, *3.4* and *3.5*) from the pollen of *C. japonica*. Then, one-month-old *C. japonica* seedlings were treated with an aqueous solution of 200 mM NaCl or plant hormones (salicylic acid (SA) and abscisic acid (ABA)) for 24 h, and it was found that salt stress induced expression of *Cryj 3.1* and *Cryj 3.4*, SA induced expression of *Cryj 3.4*, and ABA weakly induced expression of *Cryj 3.5*, indicating that various treatments can also promote allergen effects [20]. Although most studies on the effects of stress focus on morphology and physiology [16,17], some molecular studies of *C. fortunei* have also been reported, and they have all focused on the identification of differentially expressed genes and on the functional verification of genes [21,22]. Among the results, the gene expression profile is extremely important, and in gene expression analysis, the most commonly used internal RGs can no longer meet the requirements of accurate quantification. Therefore, it is necessary to study the internal RGs of *C. fortunei*.

We selected 12 candidate RGs, namely, *18S, actin, cyclophilin* (*CYP*), *histone H4* (*HIS4*), *heat shock protein 70* (*HSP70*), *serine/threonine-protein kinase* (*PBL*), *phosphoglycerate kinase 1* (*PGK1*), *protein phosphatase 2A* (*PP2A*), *large subunit of the ribulose-1,5-bisphosphate carboxylase/oxygenase* (*rbcl*), *60S ribosomal protein L2* (*RPL2*), *tubulin α-2* (*TUA2*), *and ubiquitin-conjugating enzyme* (*UBC*), and systematically studied and analyzed the expression stability of these genes under abiotic stress (including low/high temperature, drought, and salinity) and various hormone treatments (methyl jasmonate (MeJA), ABA, SA, and gibberellin (GA_3_)), as well as in different tissues (stems, tender needles, needles, cones, and seeds), using four algorithms (delta Ct, geNorm, NormFinder, and BestKeeper) by qRT-PCR analysis. Then, the geometric mean of rankings among these four algorithms and the RefFinder network program were used to comprehensively and accurately determine the most stable RGs of *C. fortunei*. In addition, the most stable and unstable genes were used to normalize the expression levels of the three target genes, namely, *catalase* (*CAT*) and *mitogen-activated protein kinase 1/6* (*MAPK1/6*), to verify the availability of the selected RGs in different tissues or under each treatment. These results identified appropriate RGs, which can be used to normalize the expression of genes in *C. fortunei*, providing a basis for normalizing gene expression in other coniferous species.

## 2. Materials and Methods

### 2.1. Plant Materials and Treatments

*C. fortunei* trees from Dagangshan, Jiangxi Province, China, exhibiting good growth with no disease or insect pests, were selected as the mother trees. In June 2019, semi-lignified branches with 2–3 lateral buds were cut as cuttings (12–16 cm). These cuttings with flat cuts on the top and 45° oblique cuts on the bottom were soaked in distilled water for 12 h, followed by rinsing with distilled water 3 times after surface sterilization with 1% (*m*/*v*) calcium hypochlorite (Ca(ClO)_2_) for 10 min and then by soaking with 0.1 g L^−1^ GGR rooting powder (Beijing Aibiti Biological Technology Co., Ltd., Beijing, China) for 4 h. The treated *C. fortunei* cuttings were placed in round plastic planting pots (15 cm in diameter and 15 cm height) with mixed soil substrate (peat:perlite:vermiculite:yellow sand, 1/1/1/1, *v*/*v*/*v*/*v*). These cutting seedlings were placed in the greenhouse of the Baima Teaching and Research Base of Nanjing Forestry University (31°37′ N, 119°11′ E), Nanjing, Jiangsu Province, China, with a 12/12-h photoperiod (day/night).

In September 2020, 123 *C. fortunei* plants with similar growth states were selected for 8 stress experiments. For abiotic stress, the *C. fortunei* cutting seedlings were exposed to 4 and 42 °C to simulate low- and high-temperature stresses, respectively; the plants were treated with 15% (*m*/*v*) polyethylene glycol (PEG)-6000 and 200 mM sodium chloride (NaCl) in 1/4-strength Hoagland solution (200 mL per plant) to simulate drought and salt stress, respectively [1,4]. For hormone treatments, the plants were sprayed evenly with 200 μM SA, 200 μM MeJA, 200 μM ABA, and 200 μM GA_3_ until all needles were completely moistened (150 mL per plant) [1,4]. The plants, except for those under low- or high-temperature stress, were cultivated at 25 °C, and all plants were cultivated in a light incubator (MLR 351H, Sanyo Electric Co., Ltd., Osaka, Japan) with the same photoperiod (12-h light/12-h dark cycle) and 60% relative humidity. Three biological replicates were performed for each treatment at each treatment timepoint (5 × 3 plants), and samples were taken at 0, 2, 6, 12, 24, and 48 h under each prolonged stress treatment [1]. Different tissue samples (stems, tender needles, needles, cones, and seeds) were collected from *C. fortunei* plants grown in a natural environment for 15 years. After the samples were collected, they were quickly frozen in liquid nitrogen and then stored at −80 °C until RNA extraction.

### 2.2. RNA Extraction and cDNA Preparation

Total RNA was extracted from the samples (0.1 g) using an RNAprep Pure Plant Kit (Polysaccharides/Polyphenolics-Rich) (Bioteke Co., Beijing, China) according to the manufacturer’s instructions. The RNA concentration and integrity were measured with a spectrophotometer (NanoDrop 2000, Thermo Scientific, Wilmington, DE, USA) and 1% (*w*/*v*) agarose gel electrophoresis, respectively. After quality verification, 0.8 μg of each total RNA sample were reverse transcribed with the HiScript III RT SuperMix Kit (Vazyme Biotech Co., Ltd., Nanjing, Jiangsu, China) according to the manufacturer’s protocol and then stored in a freezer at −20 °C until further use.

### 2.3. Selection of Candidate RGs and Primer Design

The transcriptome database of *C. fortunei* needles in our laboratory was configured into the local database of BioEdit software (Micro Focus International Ltd., Rockville, England) [23]. The unigene sequences in the transcriptome data were compared according to the commonly used internal RG sequences in previous reports, and 12 unigenes with high similarity and consistent annotation information in the local *C. fortunei* database were selected. BLASTX was performed against the NCBI non-redundant (NR) database to conduct online analysis of the conserved domains and open reading frames (ORFs) of the genes, and Primer Premier 5.0 software (Premier Biosoft International, Palo Alto, CA, USA) was used to design the RG primers based on these coding sequences (CDSs). The parameters were set as follows: PCR product length was 70–250 bp, dissolution temperature was 58–62 °C, and GC content was 40–60%. Then, we used NCBI Primer-BLAST (http://www.ncbi.nlm.nih.gov/tools/primer-blast/, accessed on 7 September 2012) for the specific detection of plant primers. Finally, we identified 12 genes as candidate RGs, and all primers (Table 1) were synthesized by Tsingke Biotech Co., Ltd. (Nanjing, Jiangsu Province, China).

### 2.4. RT-PCR and qRT-PCR Analysis

RT-PCR amplification was performed using the Fast PCR Kit (Vazyme Biotechnology Co., Ltd., Nanjing, Jiangsu Province, China) and the reaction system was set as follows: 2 μL of each primer pair (10 μM forward and reverse primers), 1 µL of cDNA, 10 µL of 2 × Rapid Taq Master Mix, and 7 µL of ddH_2_O. The amplification program was set as follows: 95 °C for 3 min, followed by 40 cycles of 95 °C for 15 s, 60 °C for 15 s and 72 °C for 15 s, and finally extension at 72 °C for 5 min. The accuracy of the designed primers was verified by 2.5% (*w*/*v*) agarose gel electrophoresis after PCR.

Two microliters of each cDNA template for all samples were mixed evenly, and the cDNA was serially diluted (1:4, 1:24, 1:124, 1:624, 1:3124, 1:15624; cDNA:water, *v*:*v*). The Ct value at each concentration was determined to establish a standard curve, and the correlation coefficient (R^2^) and PCR efficiency (E%) were calculated [24].

All qRT-PCRs were carried out by using the ChamQTM SYBR^®^ qPCR Master Mix Kit (Low ROX Premixed) (Vazyme Biotechnology Co., Nanjing, China), and the reaction system (20 µL) was as follows: 10 µL of 2 × ChamQ SYBR qPCR Master Mix (Low ROX Premixed); 2 µL of 5-fold-diluted cDNA (cDNA:water, 1:4, *v*:*v*); 0.8 µL of each specific primer pair (10 μM forward and reverse primers); and 7.2 µL of ddH_2_O. In addition, for each gene, a non-template control was included. qRT-PCR was performed on an Applied Biosystems (ABI) 7500 fast real-time PCR system (ABI, Foster City, CA, USA) for amplification, and the procedure was as follows: 30 s at 95 °C, followed by 40 cycles of 95 °C for 10 s, and 60 °C for 30 s; then a melting curve was generated at 60–95 °C immediately after completion of the qRT-PCR to detect primer dimerization and other artefacts of amplification. Each reaction had three biological replicates and three technical replicates.

### 2.5. Gene Expression Stability Analysis

Four different algorithms, i.e., delta Ct [25], geNorm (version 3.5) [26], NormFinder (version 0.953) [27], and BestKeeper (version 1.0) [28], were used to carry out statistical analysis on the stability of the expression of RGs in different samples. For geNorm and NormFinder, the original Ct value was converted to 2^−ΔCt^ (delta Ct = original Ct value − lowest Ct value in each group) and then used for the stability analysis of RGs, while for BestKeeper, the E value calculated by the LinRegPCR program based on the original Ct value and the original Ct value were used to calculate the coefficient of variation (CV) and standard deviation (SD) of the candidate RG expressions. In addition, geNorm can also determine the appropriate internal RG number by calculating the paired difference value V_n_/V_n+1_ of two consecutive normalization factors.

The geometric mean was the average of the rankings of genes in the four algorithms (delta Ct, geNorm, NormFinder, and BestKeeper) under each treatment, in different tissues or across all samples. At the same time, RefFinder analysis (https://www.heartcure.com.au/reffinder/, accessed on 31 January 2012) was also used to comprehensively verify results of RG stability analysis.

### 2.6. Validation of RGs by qRT-PCR

To examine the reliability of the selected RGs, the optimal RGs and the least stable RGs were used to normalize the expression levels of three target genes (*CAT*, *MAPK1*, and *MAPK6*) using the 2^−ΔΔCt^ method [29] under each experimental condition. Primer pairs for amplification of *CAT* (forward: 5′–TGATGTGGGTATCCCGTTGA–3′ and reverse: 5′–GGTTGCGTGACTATGATTCGTT–3′), *MAPK1* (forward: 5′–TTGATGACCAAATATGGGACG–3′ and reverse: 5′–CAACGGTTTGCATTGTTGC–3′), and *MAPK6* (forward: 5′–GAGGCTGATCTTGGATTTGTTC–3′ and reverse: 5′–CAGGCTGGCTCATCACTCAC–3′) were designed.

## 3. Results

### 3.1. Assessment of Primer Specificity and PCR Efficiency

A total of 12 candidate RGs were selected for gene normalization studies (Table 1). To determine the primer specificity of each primer, 2.5% (*w*/*v*) agarose gel electrophoresis and melting curve analysis were performed, and the results showed that we obtained specific target fragments with the expected lengths and sequences of these genes (Supplementary Figure S1). The qRT-PCR efficiency ranged from 95.14% to 109.94%, and the R^2^ of all primer pairs ranged from 0.980 to 0.997 (Table 1). These results showed that all 12 pairs of primers met the requirements of qRT-PCR and could be used in further analysis.

### 3.2. Expression Profiles of Candidate RGs

**Figure 1:**
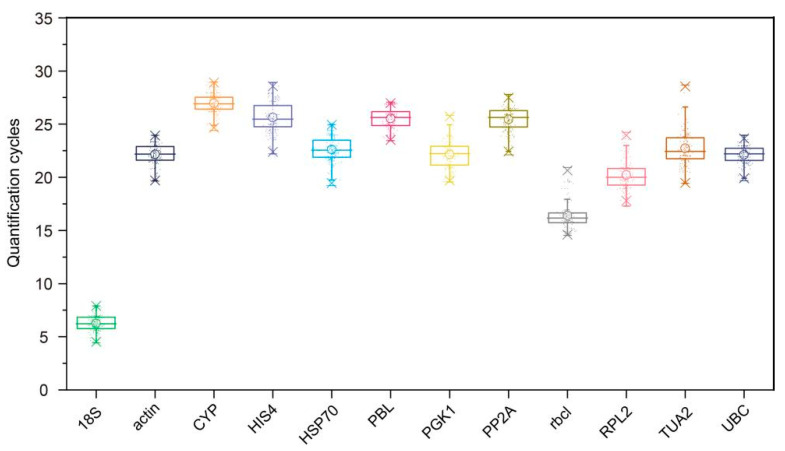
Quantification cycle values of 12 candidate reference genes in 159 samples. The boxes indicate the 25th and 75th percentiles, with the lines in the center of the boxes representing the medians. The whiskers and asterisks represent the 99% confidence intervals and outliers, respectively. The upper and lower horizontal lines indicate the maximum and minimum values, respectively, and the small circles represent the average values.

To investigate the applicability of 12 candidate genes, we analyzed the expression levels (Ct values) of these RGs in all 159 samples (including under abiotic stress (heat, cold, drought, and salinity), under hormone treatments (ABA, SA, MeJA, and GA_3_) and in different tissues (stem, seeds, cone, leaves, and tender leaves)) by qRT-PCR analysis. These average Ct values varied from 6.241 (for *18S*) to 26.958 (for *CYP*), among which *18S* and *rbcl* had relatively low Ct values, indicating that they had a high initial copy number in the samples and greater expression levels, whereas *HIS4*, *PP2A*, *PBL*, and *CYP* had the lowest expression levels (Figure 1). Moreover, the *18S* expression levels were the least variable (3.011 Ct, the maximum and minimum Ct values were 7.629 and 4.619, respectively) among all samples, followed by *PBL* (3.610 Ct), whereas the *TUA2* expression levels showed the greatest variability (8.787 Ct, ranging from 19.601 to 28.388) (Figure 1). Apparently, *18S* and *PBL* can be used as two stable RGs, but further analysis of these genes is needed.

### 3.3. Expression Stability of Candidate RGs

Four different algorithms, including delta Ct, geNorm, NormFinder, and BestKeeper, were used to estimate the expression stability of the candidate RGs under various experimental conditions.

#### 3.3.1. Delta Ct Analysis

**Figure 2:**
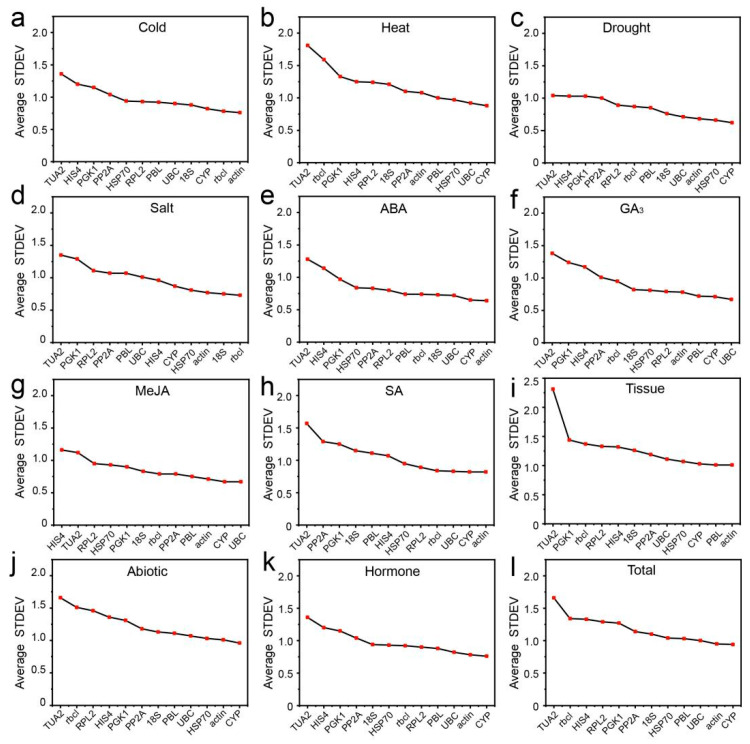
Average standard deviation (STDEV) indicated by delta Ct analysis. (**a**) 4 °C cold stress; (**b**) 42 °C heat stress; (**c**) drought stress simulated by 15% PEG-6000 treatment; (**d**) salt stress stimulated by 200 mM NaCl treatment; (**e**) 200 μM ABA treatment; (**f**) 200 μM GA_3_ treatment; (**g**) 200 μM MeJA treatment; (**h**) 200 μM SA treatment; (**i**) in different tissues (stems, tender needles, needles, cones, and seeds); (**j**) under abiotic stress; (**k**) under hormone treatments; and (**l**) in total samples.

The delta Ct method is used to compare the relative expression levels of “gene pairs” in groups of samples and ranks the stability of candidate RGs based on the reproducibility of the average standard deviation (STDEV) of gene expression differences between samples [25]. The lower the STDEV value is, the more stable the gene. As shown in Figure 2, under cold stress, *actin* and *rbcl* had the smallest STDEVs of 0.81 and 0.82, respectively, and were the most stable genes (Figure 2a), whereas in heat-, GA_3_-, or MeJA-treated samples, *CYP* and *UBC* were the most stable genes (Figure 2b,f,g). Under the drought and salt treatments, *CYP* and *HSP70*, and *rbcl* and *18S* were the two most stable RGs (Figure 2c,d). In tissues, *PBL* and *actin* showed the highest stability (Figure 2i). In addition, under ABA treatment, SA treatment, abiotic stress, hormone treatments, and all sample conditions, *CYP* and *actin* all showed high stability (Figure 2e,h,j–l). In contrast, the statistical results for the last three rankings in each treatment/tissue showed that *TUA2* (12 times, 100.00%), *PGK1* (9 times, 75.00%) and *HIS4* (7 times, 58.33%) were the most unstable under most conditions (Figure 2).

#### 3.3.2. GeNorm Analysis

**Figure 3:**
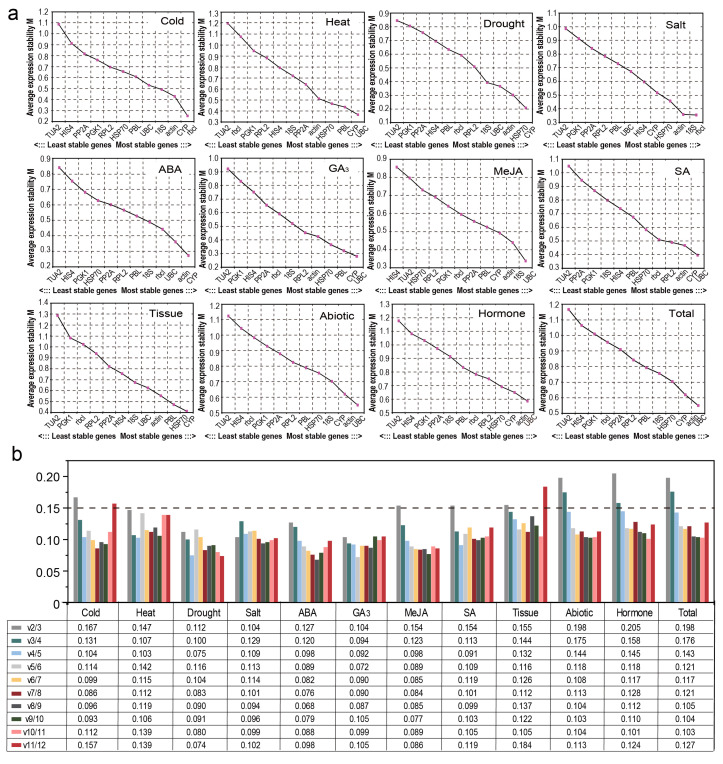
Average expression stability values and pairwise variations indicated by geNorm analysis. (**a**) The expression stability values (M) and rankings of the 12 candidate reference genes of *Cryptomeria fortunei* calculated using geNorm. The most and least stable genes are on the right and left, respectively. (**b**) Optimal number of reference genes determined for *C. fortunei*.

GeNorm software can calculate the stability (M value) of each RG to evaluate stability and 1.5 is the default threshold of M; the smaller the M value is, the better the stability of the RG [26]. For all samples, under all hormone treatments or under abiotic stress, *UBC* and actin were the most stable genes; for each treatment, there were significant differences in the expression stability of the 12 genes (Figure 3a). More specifically, *rbcl* and *CYP* were the most stably expressed genes under cold stress; under drought stress simulated by PEG treatment or in various tissues, the most stable genes with standardized data were *CYP* and *HSP70*; and the samples treated with NaCl, *rbcl*, and *18S* had higher expression stability than the other genes. For ABA-treated samples, *CYP* and *actin* were more stable than the other genes, and *UBC* and *18S* showed the strongest stability under MeJA treatment. *UBC* and *CYP* showed the most stable expression levels under SA treatment, GA_3_ treatment, or for samples treated at 42 °C. In most cases, *TUA2*, *PGK1*, and *HIS4* were generally the least stable RGs (Figure 3a).

A single RG usually does not meet the stability requirements for standardization, so two or more RGs are needed to reduce errors and obtain more accurate quantification of target gene expression. GeNorm is based on pairwise changes (V_n_/V_n+1_), using a threshold of 0.15 to determine the optimal number of RGs for each treatment [26]. As shown in Figure 3b, under a single abiotic stress (heat, drought, or salinity) or under ABA or GA_3_ treatment, the pairwise variation values V_2_/V_3_ were all less than 0.15, indicating that two suitable RGs were sufficient to standardize these treatment data, whereas under cold, MeJA, and SA treatments and in different tissues, the V_3_/_4_ values were all less than 0.15, indicating that three RGs were required. Under abiotic stress and various hormone treatments and after merging all samples, the V_4_/_5_ values were 0.144, 0.145, and 0.143, respectively, indicating that four RGs were necessary.

#### 3.3.3. NormFinder Analysis

We also calculated the stability values using NormFinder to evaluate the stability of the expression of these 12 candidate RGs, and low stability values indicated high expression stability [27]. The most stable genes were as follows: *CYP* and *HSP70* under drought stress; *actin* and *UBC* under hormone treatments; *CYP* and *UBC* under heat/MeJA/SA/GA_3_/abiotic stress and in the total samples; *rbcl* and *CYP*, *rbcl* and *18S*, and *actin* and *CYP* were the top two most stable genes under cold, salt, and ABA treatment, respectively. *Actin*, *PBL*, and *HSP70* showed high expression stability in tissues (Table 2). In addition, *HIS4*, *PGK1*, and *TUA2* were the least stable genes in all samples/treatments (Table 2). Notably, despite the differences in rankings between geNorm and NormFinder, the five most stable genes as well as the three most unstable genes in all samples were basically the same (Figure 3a, Table 2).

#### 3.3.4. BestKeeper Analysis

BestKeeper ranks RGs based on the CV and SD of the average Cq value in the qRT-PCR analysis [28]. The most stable genes showed the lowest SD ± CV value, and the SD values were also less than 1. The stability of *UBC* ranked first under the MeJA and SA treatments, with SD ± CV values of 0.35 ± 1.59 and 0.35 ± 1.54, respectively (Table 3). *CYP* (0.35 ± 1.29) was the best RG under cold stress; *PP2A* (0.42 ± 1.62) ranked first under heat stress; *actin* (0.33 ± 1.52) ranked first under PEG treatment; *rbcl* ranked first under the ABA and hormone treatments, with SD ± CV values of 0.37 ± 2.26 and 0.50 ± 3.05, respectively (Table 3). Although *18S* ranked first under salinity, GA_3_ treatment, and abiotic stress and in all tissues and samples, with SD ± CV values of 0.38 ± 6.18, 0.46 ± 7.20, 0.47 ± 7.50, 0.68 ± 11.98, and 0.51 ± 8.10, respectively, its CV values were often the highest and were significantly larger than those of the other genes (Table 3). Therefore, *rbcl* (0.39 ± 2.45), *CYP* (0.47 ± 1.76), and *UBC* may be the most stable genes under salt stress, under GA_3_ treatment, and under abiotic stress/in all tissues/in all samples, respectively (Table 3). BestKeeper suggested that *18S*, *UBC*, *actin*, and *CYP* were the most stable RGs for merging the data of all samples (Table 3). At the same time, we analyzed the genes with SD values ≥ 1 and found that *TUA2*, *HIS4*, and *PGK1* were the least stable RGs (Supplementary Table S1).

### 3.4. Comprehensive Stability Analysis of the RGs

**Figure 4:**
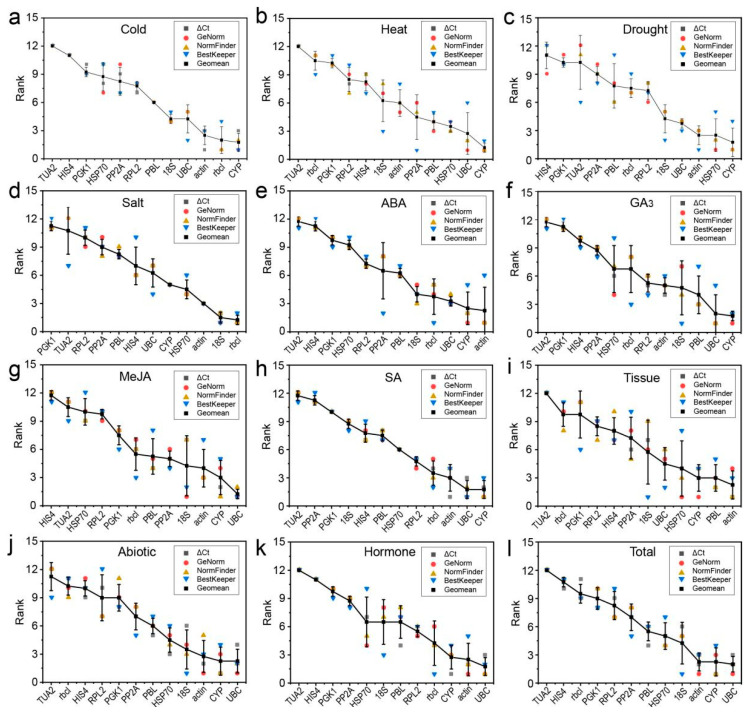
Comprehensive rankings of the 12 RGs in *C. fortunei* calculated as the geometric mean of the four types of rankings for each group of samples. (**a**) 4 °C cold stress; (**b**) 42 °C heat stress; (**c**) drought stress simulated by 15% PEG-6000 treatment; (**d**) salt stress simulated by 200 mM NaCl treatment; (**e**) 200 μM ABA treatment; (**f**) 200 μM GA_3_ treatment; (**g**) 200 μM MeJA treatment; (**h**) 200 μM SA treatment; (**i**) in different tissues (stems, tender needles, needles, cones, and seeds); (**j**) under abiotic stress; (**k**) under hormone treatments; in (**l**) in total samples.

To reduce the influence of the limitations and deviations of a single algorithm, the stability of the RGs was analyzed by using the geometric mean of four algorithms (delta Ct, geNorm, NormFinder, and BestKeeper) to determine the best RGs. The comprehensive RG ranking results are shown in Figure 4. Under cold stress, *rbcl*, *CYP*, and *actin* was the best combination; under drought stress, *HSP70* and *CYP* were the most stable RGs; under high-salinity stress, *rbcl* and *18S* rRNA exhibited the highest stability; under ABA treatment, *CYP* and *actin* showed the highest stability; and *PBL*, *actin*, and *CYP* were the most stable RGs in different tissues. *CYP* and *UBC* showed the highest stability under heat stress, under GA_3_ treatment, under MeJA/SA treatment (also including *actin*), under hormone treatments (also including *actin* and *rbcl*), and under abiotic stress/in all samples (also including *actin* and *18S*).

**Figure 5:**
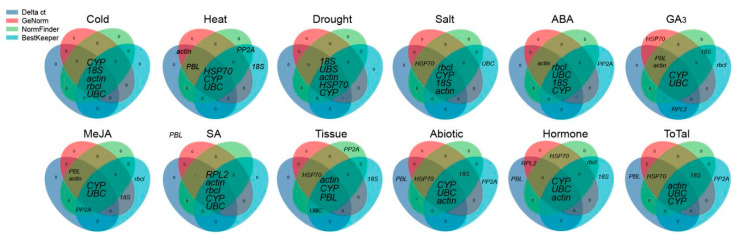
The 5 most stable reference genes (RGs) indicated by delta Ct analysis, geNorm, NormFinder, and BestKeeper. The blue, pink, green, and sky-blue circles each contain the 5 most stable RGs determined by delta Ct analysis, geNorm, NormFinder, and BestKeeper, respectively. The genes in the overlapping area are those confirmed as the 5 most stable RGs by more than one algorithm.

We further used the RefFinder program to comprehensively verify the rankings of candidate RGs, and the results were basically the same as those for the geometric mean, with only slight differences under MeJA-treated samples. For example, *UBC*, *CYP*, and *18S* were the best RGs under MeJA stress, followed by *actin*. In addition, these stable RGs selected under various experimental conditions were basically the five most stable genes selected by the four algorithms (or at least two, for example, geNorm and BestKeeper) (Figure 5). By contrast, we conducted statistical analysis of the three most unstable RGs determined by the two comprehensive evaluation methods, and the results showed that *TUA2*, *PGK1*, and *HIS4* were the least stable RGs (Supplementary Table S2).

### 3.5. Validation of the Stability of RGs

To verify the accuracy of the stable expression of RGs, three genes (*CAT*, *MAPK1*, and *MAPK6*) were selected as target genes. The expression of the antioxidant *CAT* gene is induced by many abiotic stress factors, including cold, heat, drought, and salinity [30]; mitogen-activated protein kinases (*MAPK1* and *MAPK6*) are important transmitters of signals from the cell surface to the inside of the nucleus and play an important role in the regulation of plant growth and development and abiotic stress resistance and tolerance [31,32,33]. We combined the geNorm pairwise change (V_n_/V_n+1_) results, selected multiple best and the worst RGs according to the comprehensive ranking results for each treatment/tissue, and used these RGs to standardize gene expression data.

**Figure 6:**
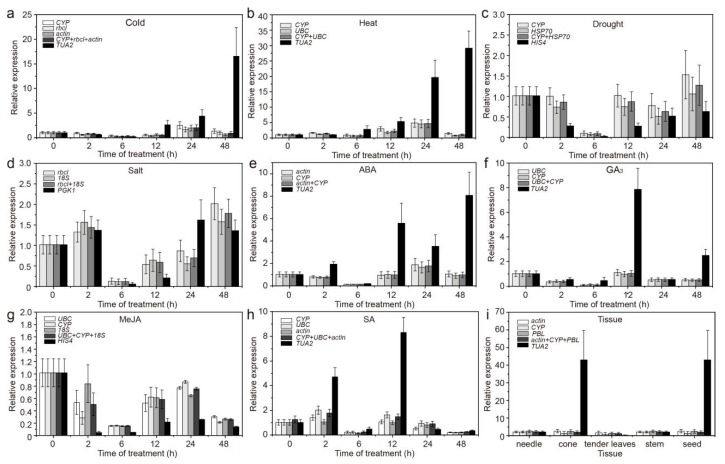
Relative expression levels of *CAT* under different experimental conditions normalized to the top two/three stable genes and the least stable genes. (**a**) 4 °C cold stress; (**b**) 42 °C heat stress; (**c**) drought stress simulated by 15% PEG-6000 treatment; (**d**) salt stress simulated by 200 mM NaCl treatment; (**e**) 200 μM ABA treatment; (**f**) 200 μM GA_3_ treatment; (**g**) 200 μM MeJA treatment; (**h**) 200 μM SA treatment; and (**i**) in different tissues (stems, tender needles, needles, cones, and seeds). The error bars represent standard deviations (SDs) (*n* = 3).

**Figure 7:**
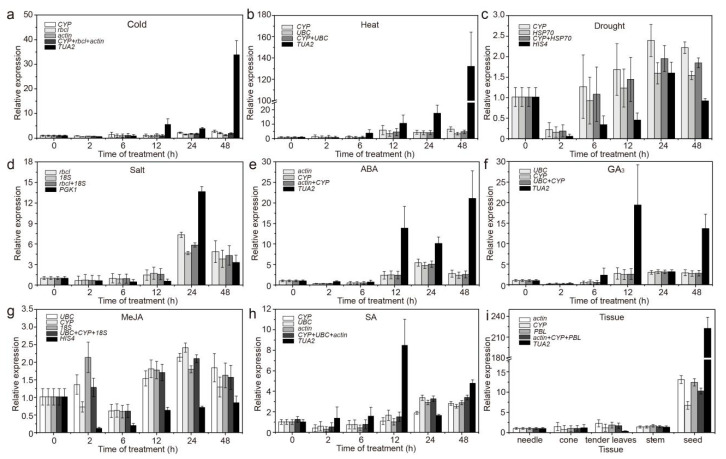
Relative expression levels of *MAPK1* under different experimental conditions normalized to the top two/three most stable genes and the least stable genes. (**a**) 4 °C cold stress; (**b**) 42 °C heat stress; (**c**) drought stress simulated by 15% PEG-6000 treatment; (**d**) salt stress simulated by 200 mM NaCl treatment; (**e**) 200 μM ABA treatment; (**f**) 200 μM GA_3_ treatment; (**g**) 200 μM MeJA treatment; (**h**) 200 μM SA treatment; and (**i**) in different tissues (stems, tender needles, needles, cones, and seeds). The error bars represent standard deviations (SD) (*n* = 3).

**Figure 8:**
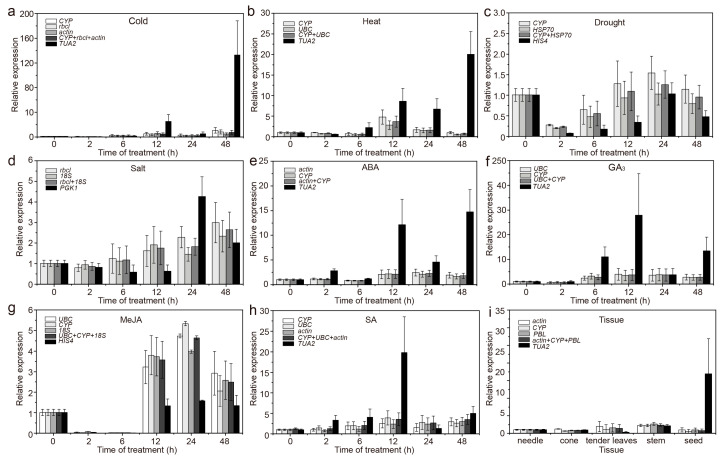
Relative expression levels of *MAPK6* under different experimental conditions normalized to the top two/three most stable genes and the least stable genes. (**a**) 4 °C cold stress; (**b**) 42 °C heat stress; (**c**) drought stress simulated by 15% PEG-6000 treatment; (**d**) salt stress simulated by 200 mM NaCl treatment; (**e**) 200 μM ABA treatment; (**f**) 200 μM GA_3_ treatment; (**g**) 200 μM MeJA treatment; (**h**) 200 μM SA treatment; and (**i**) in different tissues (stems, tender needles, needles, cones, and seeds). The error bars represent standard deviations (SD) (*n* = 3).

In different tissues, the expression patterns obtained when using the most and the least stable RGs under specific experimental conditions were different. More specifically, using stable RGs, *CAT* and *MAPK6* showed the highest expression (<3 times, compared to needles) in stems, whereas using an unstable internal control, the expression of *CAT* and *MAPK6* was highest in seeds (15–42 times higher than that in needles), and the expression increased significantly (Figure 6i and Figure 8i). In addition, when using stable and unstable RGs, although *MAPK1* was the most highly expressed in seeds, the difference was more than 20-fold (Figure 7i).

Under different treatments, when using different stable RGs, *CAT*, *MAPK1*, and *MAPK6* showed similar expression patterns, but their expression levels differed between treatments (Figure 6, Figure 7 and Figure 8). More specifically, under abiotic stress, such as cold and heat, *CAT*, *MAPK1*, and *MAPK6* were upregulated or downregulated when using stable RGs, but the magnitude was low; when the most unstable RGs were used for normalization, the expression of *CAT*, *MAPK1*, and *MAPK6* fluctuated significantly. Overall, the results show that if different RGs are used to correct the expression of target genes in qRT-PCR analysis, different results will be obtained. If the RGs are incorrectly selected, the relative expression levels of the target genes may be incorrectly estimated.

## 4. Discussion

qRT-PCR has been widely used in the study of gene expression in recent years because of its rapidness and high sensitivity, accuracy, and reproducibility [1,2,3,4]. The key to obtaining correct gene expression results is to select the appropriate RGs to standardize the data. Improper selection of RGs may lead to incorrect experimental conclusions. However, there is no report of systematic selection of suitable RGs of *C. fortunei* for qRT-PCR analysis.

In this study, we selected 12 candidate RGs, most of which have been studied in different plants, such as Masson pine (*Pinus massoniana*) [15], cortex eucommiae (*Eucommia ulmoides*) [34], and soybean (*Glycine max*) [2]. In terms of standardization and quality, the PCR efficiency of candidate RG primer pairs ranged from 95.14% to 109.94%, and the R^2^ of linear amplification ranged from 0.980 to 0.997 (Table 1). These results indicate that the primer pairs used for these RGs have high accuracy, efficiency, and sensitivity. The average Ct values of candidate RGs varied from 6.241 (*18S*) to 26.958 (for *CYP*) (Figure 1). Regarding Chinese tulip trees (*Liriodendron chinense*) [14] and *E. ulmoides* [34], the candidate RGs also exhibited different expression levels in the test materials, and the average Ct values of candidate RGs ranged between 17 and 29. These results indicate that in a given whole sample, the expression levels of 12 RGs were in a wide range, and none of the RGs had constant expression levels in different samples. Therefore, it is necessary to select suitable RGs for gene standardization under specific experimental conditions before selecting RGs as an internal control for qRT-PCR analysis.

To date, many algorithms have been widely used for screening and evaluating RG stability. In this study, four algorithms (delta Ct, NormFinder, geNorm, and Bestkeeper) were used to analyze the expression of 12 RGs in different tissue samples or under different abiotic stress/hormone treatments in *C. fortunei*. We found that the top five genes selected by various algorithms were generally similar (Figure 5). For example, in all samples, the top five genes of the four algorithms all included *UBC*, *actin*, and *CYP*; in addition, the genes selected by GeNorm (NormFinder), delta Ct, and BestKeeper also included *18S* and *HSP70*, *PBL* and *HSP70*, and *18S* and *PP2A*, respectively. However, there were indeed some differences between the stability analysis results obtained using different algorithms (Figure 2 and Figure 3 and Table 2 and Table 3). For example, in all samples, GeNorm and NormFinder analyses indicated that *UBC*, *actin*, *CYP*, and *HSP70* were the most stable genes; delta Ct thought that *CYP*, *actin*, *UBC*, and *PBL* showed the highest stability; while BestKeeper showed that *18S*, *UBC*, *actin*, and *CYP* performed better than the other genes. This phenomenon has also been observed in studies on RGs in plants, such as citrus (*Citrus reticulata*) [35], *L. chinense* [14], and wild barley (*Hordeum brevisubulatum*) [4]. It is speculated that each algorithm has different principles and methods for evaluating stability, so the final conclusions are also different.

In the actual application process, it is necessary to comprehensively consider the analysis results provided by delta Ct, NormFinder, GeNorm, and BestKeeper [4,36,37]. To avoid differences caused by the software, the geometric mean method or RefFinder can be used to comprehensively analyze the results obtained with different software when selecting RGs, and finally, the optimal RGs can be obtained. Fortunately, we found that the results of the geometric mean method for each treatment/tissue were similar to the RefFinder results (Figure 4 and Supplementary Table S3), indicating that under cold stress, *rbcl*, *CYP*, and *actin* was the best combination; under drought, high-salinity stress, or under ABA treatment, *HSP70* and *CYP*, *rbcl* and *18S*, or *CYP* and *actin* were the most stable RGs; and *PBL*, *actin*, and *CYP* were the most stable RGs in different tissues. *CYP* and *UBC* showed the highest stability under heat stress, under GA_3_ treatment, under SA treatment (also including *actin*), under MeJA treatment (also including *18S*), under hormone treatments (also including *actin* and *rbcl*), and under abiotic stress/in all samples (also including *actin* and *18S*). There were also certain differences in the optimal combination of RGs under different treatments/in different tissues of the same species. Similar results were also obtained via research on differences in RGs in *H.* *hamabo* [1], *E. ulmoides* [34], *P. massoniana* [15], and other plants.

Although no RG was consistently the best under the various experimental conditions, *CYP*, *UBC*, *actin*, and *18S* were proposed as good choices for studying gene expression in *C. fortunei*, whereas *TUA2*, *PGK1*, and *HIS4* were the least stable RGs. This is similar to the results for RGs in other plants. For example, in the evaluation of the stability of RGs in different tissues of *P. miliaceum* and under stress treatments, it was found that *18S* was suitable as an RG [12]; *actin* was determined to be the most suitable internal control gene in *P. massoniana* by qRT-PCR analysis under stress (salinity, drought, cold, and heat) [15] and was the most stably expressed RG in *H. hamabo* in all samples (in different tissues and under abiotic stress) [1]; *CYP* was a stable RG under abiotic stress in pineapple (*Ananas comosus*) [38]; and *UBC* was the stable RG for the groups “natural growth”, “abiotic stress”, and “total” in *E. ulmoides* [34]. However, there were some differences in RGs among species; for example, *TUA* was the most stable RG in all processed samples of *P.* *massoniana* [15]. In a study of *Nitraria tangutorum*, *HIS* was suitable for normalization in different organs, under abiotic stress and hormone stimuli [39]. These results are verified by the fact that RGs exhibit species-specific expression.

To compare normalization results, the best and the worst ranked genes were used for normalization of *CAT*, *MAPK1*, and *MAPK6* expression. Under various stresses or in different tissues, inappropriate candidate RGs were used, resulting in incorrect estimation of the expression (underestimation/overestimation) or expression trends (Figure 6, Figure 7 and Figure 8), emphasizing that the use of unsuitable RGs could lead to unexpected and uncertain results. This study screened only 12 commonly used RGs and selected samples of *C. fortunei*, that is, a sample of different tissues and samples under eight treatments (hormone treatments and abiotic stress). Follow-up research should expand the screening range of RGs and develop new RGs that are suitable for the experimental system to provide a theoretical basis for the development and application of RGs of *C. fortunei* and aid molecular biological research on *C. fortunei*.

## 5. Conclusions

Taken together, for the first time, this study systematically and comprehensively screened the best RGs for *C. fortunei* under different abiotic stress factors, under hormone treatments, and in different tissues. The results indicate that the following genes could be used as RGs under various experimental conditions: *CYP* and *HSP70* under drought; *CYP* and *actin* under ABA treatment; *rbcl*, *CYP*, and *actin* under cold stress; *rbcl* and *18S* under high-salinity stress; *PBL*, *actin*, and *CYP* in different tissues; and *CYP* and *UBC* showed the highest stability under heat stress, under GA_3_ treatment, under SA treatment (also including *actin*), under MeJA treatment (also including *18S*), under hormone treatments (also including *actin* and *rbcl*), and under abiotic stress/in all samples (also including *actin* and *18S*). Although no RG was consistently the best under various experimental conditions, *CYP*, *UBC*, *actin*, and *18S* were proposed as good choices for studying gene expression in *C. fortunei*. These suitable RGs will help to improve the accuracy of gene expression analysis of *C. fortunei* and aid further research on stress ecology and gene functions of *C. fortunei*.

## Figures and Tables

**Table 1:** Twelve candidate reference genes and primer sequences used in this study.

Gene Symbol	Gene Name	Primer Sequence (5′ to 3′)	Amplicon Size (bp)	PCR Efficiency (E%)	R^2^
*18S*	18S ribosomal RNA	TCTGGTCCTGTTCCGTTGG	124	100.21	0.983
		GCTTTCGCAGTGGTTCGTC			
*ACT*	actin	GTTGCCATTCAAGCCGTTCT	228	98.54	0.984
		AACAATTTCACGCTCAGCAGTAG			
*CYP*	cyclophilin	TCTCGGGCAGCATTTCACGC	79	105.98	0.990
		AGCCGAAACTGGCGCCAACA			
*HIS4*	histone H4	TTCCAGTTGGAAGGAAAAATGTCTG	253	107.83	0.981
		GGCGAGCGTGCTCAGTGTAT			
*HSP70*	heat shock protein 70	AACGCAAGGGCTTTGAGAA	139	103.40	0.980
		ACCTGGCACGGGTTATGGT			
*PBL*	serine/threonine-protein kinase	AGTTCTGCCATGGCCCGTGA	223	109.08	0.994
		TGCAGTGCCAACAACCGCTG			
*PGK1*	phosphoglycerate kinase 1	GCGGGCGAGTAAAGTGGTA	181	95.14	0.990
		GGAGATCAAATACTTAATGGTGGGT			
*PP2A*	protein phosphatase 2A	TGAAGGAGGGAGATTTGATTGA	128	107.83	0.988
		CAGTTCCGATGCACTTGGGT			
*rbcl*	large subunit of the ribulose-1,5-bisphosphate carboxylase/oxygenase	CGTATTACAGTTCGGTGGAGGG	185	101.39	0.997
		CACAAGCGGCAGCTAGTTCA			
*RPL2*	60S ribosomal protein L2	CCAGCATCGTTGTGGGAAAG	70	102.28	0.980
		GTGACCTCCTCCTCTATGTCGTAT			
*TUA2*	tubulin α-2	CTTTCCTCGCACTCGCTGTT	181	109.94	0.992
		GGTGTAGGTAGGGCGGTCAA			
*UBC*	ubiquitin-conjugating enzyme	CTCGCAGAATCATAAAGGAAACAC	180	107.09	0.981
		CCATTGGATACTCTTCAGGCAAA			

R^2^, correlation coefficient.

**Table 2:** Expression stability values in *C. fortunei* under various treatments determined with NormFinder software.

Sample		1	2	3	4	5	6	7	8	9	10	11	12
Cold	gene	*rbcl*	*CYP*	*actin*	*18S*	*UBC*	*PBL*	*PP2A*	*RPL2*	*PGK1*	*HSP70*	*HIS4*	*TUA2*
	stability	0.062	0.125	0.164	0.326	0.394	0.516	0.516	0.561	0.598	0.625	0.750	1.298
Heat	gene	*CYP*	*UBC*	*HSP70*	*PBL*	*PP2A*	*actin*	*RPL2*	*18S*	*HIS4*	*PGK1*	*rbcl*	*TUA2*
	stability	0.104	0.316	0.340	0.441	0.444	0.534	0.608	0.609	0.637	0.721	0.951	1.142
Drought	gene	*CYP*	*HSP70*	*actin*	*UBC*	*18S*	*PBL*	*rbcl*	*RPL2*	*PP2A*	*PGK1*	*TUA2*	*HIS4*
	stability	0.051	0.175	0.245	0.263	0.337	0.430	0.439	0.467	0.571	0.580	0.604	0.606
Salt	gene	*rbcl*	*18S*	*actin*	*HSP70*	*CYP*	*HIS4*	*UBC*	*PP2A*	*PBL*	*RPL2*	*PGK1*	*TUA2*
	stability	0.097	0.159	0.198	0.259	0.347	0.453	0.530	0.579	0.596	0.629	0.779	0.840
ABA	gene	*actin*	*CYP*	*18S*	*UBC*	*rbcl*	*PBL*	*RPL2*	*PP2A*	*HSP70*	*PGK1*	*HIS4*	*TUA2*
	stability	0.160	0.176	0.256	0.294	0.295	0.308	0.361	0.406	0.421	0.539	0.692	0.811
GA_3_	gene	*UBC*	*CYP*	*PBL*	*18S*	*actin*	*RPL2*	*HSP70*	*rbcl*	*PP2A*	*HIS4*	*PGK1*	*TUA2*
	stability	0.031	0.165	0.224	0.311	0.325	0.327	0.371	0.487	0.531	0.680	0.761	0.863
MeJA	gene	*CYP*	*UBC*	*actin*	*PBL*	*PP2A*	*rbcl*	*18S*	*PGK1*	*HSP70*	*RPL2*	*TUA2*	*HIS4*
	stability	0.140	0.191	0.252	0.300	0.345	0.356	0.420	0.471	0.496	0.516	0.672	0.708
SA	gene	*CYP*	*UBC*	*rbcl*	*actin*	*RPL2*	*HSP70*	*HIS4*	*PBL*	*18S*	*PGK1*	*PP2A*	*TUA2*
	stability	0.214	0.233	0.241	0.255	0.367	0.408	0.539	0.585	0.616	0.716	0.740	0.982
Tissue	gene	*actin*	*PBL*	*HSP70*	*CYP*	*PP2A*	*UBC*	*RPL2*	*rbcl*	*18S*	*HIS4*	*PGK1*	*TUA2*
	stability	0.316	0.326	0.394	0.419	0.474	0.505	0.632	0.676	0.688	0.699	0.775	1.520
Abiotic	gene	*CYP*	*UBC*	*18S*	*HSP70*	*actin*	*PBL*	*RPL2*	*PP2A*	*rbcl*	*HIS4*	*PGK1*	*TUA2*
	stability	0.203	0.395	0.395	0.448	0.486	0.517	0.558	0.598	0.700	0.718	0.722	1.016
Hormone	gene	*UBC*	*actin*	*CYP*	*rbcl*	*HSP70*	*RPL2*	*18S*	*PBL*	*PP2A*	*PGK1*	*HIS4*	*TUA2*
	stability	0.207	0.279	0.289	0.396	0.415	0.450	0.467	0.473	0.538	0.737	0.788	0.825
Total	gene	*CYP*	*UBC*	*actin*	*HSP70*	*18S*	*PBL*	*RPL2*	*PP2A*	*rbcl*	*PGK1*	*HIS4*	*TUA2*
	stability	0.281	0.345	0.386	0.439	0.478	0.518	0.547	0.571	0.598	0.734	0.752	1.040

**Table 3:** Expression stability values of candidate reference genes calculated by BestKeeper.

Sample		1	2	3	4	5	6	7	8	9	10	11	12
Cold	gene	*CYP*	*UBC*	*actin*	*rbcl*	*18S*	*PBL*	*PP2A*	*RPL2*	*PGK1*	*HSP70*	*HIS4*	*TUA2*
	SD	0.35	0.38	0.44	0.46	0.47	0.70	0.71	0.74	0.76	0.79	1.00	1.31
	CV	1.29	1.69	1.97	2.94	7.37	2.73	2.79	3.87	3.47	3.46	3.76	5.75
Heat	gene	*PP2A*	*CYP*	*18S*	*HSP70*	*PBL*	*UBC*	*HIS4*	*actin*	*rbcl*	*RPL2*	*PGK1*	*TUA2*
	SD	0.42	0.66	0.67	0.68	0.78	0.78	0.80	0.92	1.04	1.04	1.12	1.19
	CV	1.62	2.36	10.67	2.91	2.98	3.49	2.97	4.08	5.52	4.68	4.78	4.99
Drought	gene	*actin*	*18S*	*UBC*	*CYP*	*HSP70*	*TUA2*	*RPL2*	*PP2A*	*rbcl*	*PGK1*	*PBL*	*HIS4*
	SD	0.33	0.35	0.35	0.42	0.53	0.58	0.66	0.69	0.74	0.82	0.88	1.05
	CV	1.52	5.60	1.61	1.57	2.33	2.60	3.31	2.75	4.79	3.81	3.46	4.22
Salt	gene	*18S*	*rbcl*	*actin*	*UBC*	*CYP*	*HSP70*	*TUA2*	*PBL*	*PP2A*	*HIS4*	*RPL2*	*PGK1*
	SD	0.38	0.39	0.40	0.45	0.57	0.60	0.68	0.72	0.78	0.84	0.96	1.12
	CV	6.18	2.45	1.78	2.03	2.11	2.65	3.08	2.85	3.06	3.28	4.88	5.15
ABA	gene	*rbcl*	*PP2A*	*UBC*	*18S*	*CYP*	*actin*	*PBL*	*RPL2*	*PGK1*	*HSP70*	*TUA2*	*HIS4*
	SD	0.37	0.47	0.56	0.66	0.72	0.74	0.87	0.87	0.92	0.96	1.05	1.15
	CV	2.26	1.81	2.51	9.82	2.64	3.27	3.32	4.28	4.11	4.12	4.50	4.43
GA_3_	gene	*18S*	*CYP*	*rbcl*	*RPL2*	*UBC*	*actin*	*PBL*	*PP2A*	*HIS4*	*HSP70*	*TUA2*	*PGK1*
	SD	0.46	0.47	0.47	0.53	0.59	0.65	0.66	0.69	0.78	0.78	0.79	0.96
	CV	7.20	1.76	2.88	2.67	2.71	2.95	2.56	2.74	3.00	3.47	3.51	4.41
MeJA	gene	*UBC*	*18S*	*rbcl*	*PP2A*	*CYP*	*PGK1*	*actin*	*PBL*	*TUA2*	*RPL2*	*HIS4*	*HSP70*
	SD	0.35	0.36	0.45	0.61	0.63	0.65	0.69	0.70	0.92	0.95	0.95	1.09
	CV	1.59	5.73	2.72	2.41	2.33	2.88	3.12	2.75	4.11	4.69	3.80	4.90
SA	gene	*UBC*	*rbcl*	*CYP*	*actin*	*RPL2*	*HSP70*	*PBL*	*18S*	*HIS4*	*PGK1*	*TUA2*	*PP2A*
	SD	0.35	0.35	0.41	0.44	0.54	0.64	0.82	0.85	0.87	0.91	0.98	1.05
	CV	1.54	2.19	1.52	1.96	2.74	2.82	3.24	14.33	3.43	4.09	4.33	4.20
Tissue	gene	*18S*	*UBC*	*actin*	*CYP*	*PBL*	*PGK1*	*HIS4*	*HSP70*	*RPL2*	*PP2A*	*rbcl*	*TUA2*
	SD	0.68	0.70	0.92	0.95	0.97	1.10	1.11	1.22	1.22	1.25	1.50	2.39
	CV	11.98	3.34	4.39	3.69	3.94	5.15	4.58	5.75	5.92	5.06	8.84	10.33
Abiotic	gene	*18S*	*UBC*	*actin*	*CYP*	*PP2A*	*HSP70*	*PBL*	*PGK1*	*TUA2*	*HIS4*	*rbcl*	*RPL2*
	SD	0.47	0.54	0.61	0.66	0.70	0.75	0.83	1.02	1.12	1.23	1.24	1.26
	CV	7.50	2.45	2.73	2.42	2.74	3.29	3.22	4.60	4.92	4.73	7.56	6.24
Hormone	gene	*rbcl*	*UBC*	*18S*	*CYP*	*actin*	*RPL2*	*PBL*	*PP2A*	*PGK1*	*HSP70*	*HIS4*	*TUA2*
	SD	0.50	0.56	0.60	0.62	0.67	0.75	0.78	0.82	0.88	0.91	0.96	1.03
	CV	3.05	2.51	9.51	2.29	3.03	3.73	3.03	3.21	3.98	4.01	3.74	4.52
Total	gene	*18S*	*UBC*	*actin*	*CYP*	*PP2A*	*PBL*	*HSP70*	*PGK1*	*rbcl*	*RPL2*	*HIS4*	*TUA2*
	SD	0.51	0.61	0.67	0.71	0.74	0.80	0.86	0.87	0.92	0.97	1.08	1.13
	CV	8.10	2.77	3.03	2.64	2.92	3.15	3.81	3.94	5.60	4.78	4.20	4.95

## Supplementary Materials

The following are available online at https://www.mdpi.com/article/10.3390/genes12060791/s1, Figure S1: specificity of each candidate reference gene primer pair, Table S1: statistics for genes with SD values ≥ 1 under different treatments/in different tissues, Table S2: statistics for the last three RGs in the stability rankings obtained via the two comprehensive evaluation methods, Table S3: top five genes ranked by the five tools.
