# Supplementary material for: Selection and Verification of Appropriate Reference Genes for Expression Normalization in Cryptomeria fortunei under Abiotic Stress and Hormone Treatments
Source: Genes (Basel). 2021 May 21;12(6):791. doi: 10.3390/genes12060791 (PMC8224294; doi:10.3390/genes12060791)
Supplement: Supplementary file 1 [file genes-12-00791-s001.zip › genes-1220189-supplementary.pdf]

Supplementary Materials

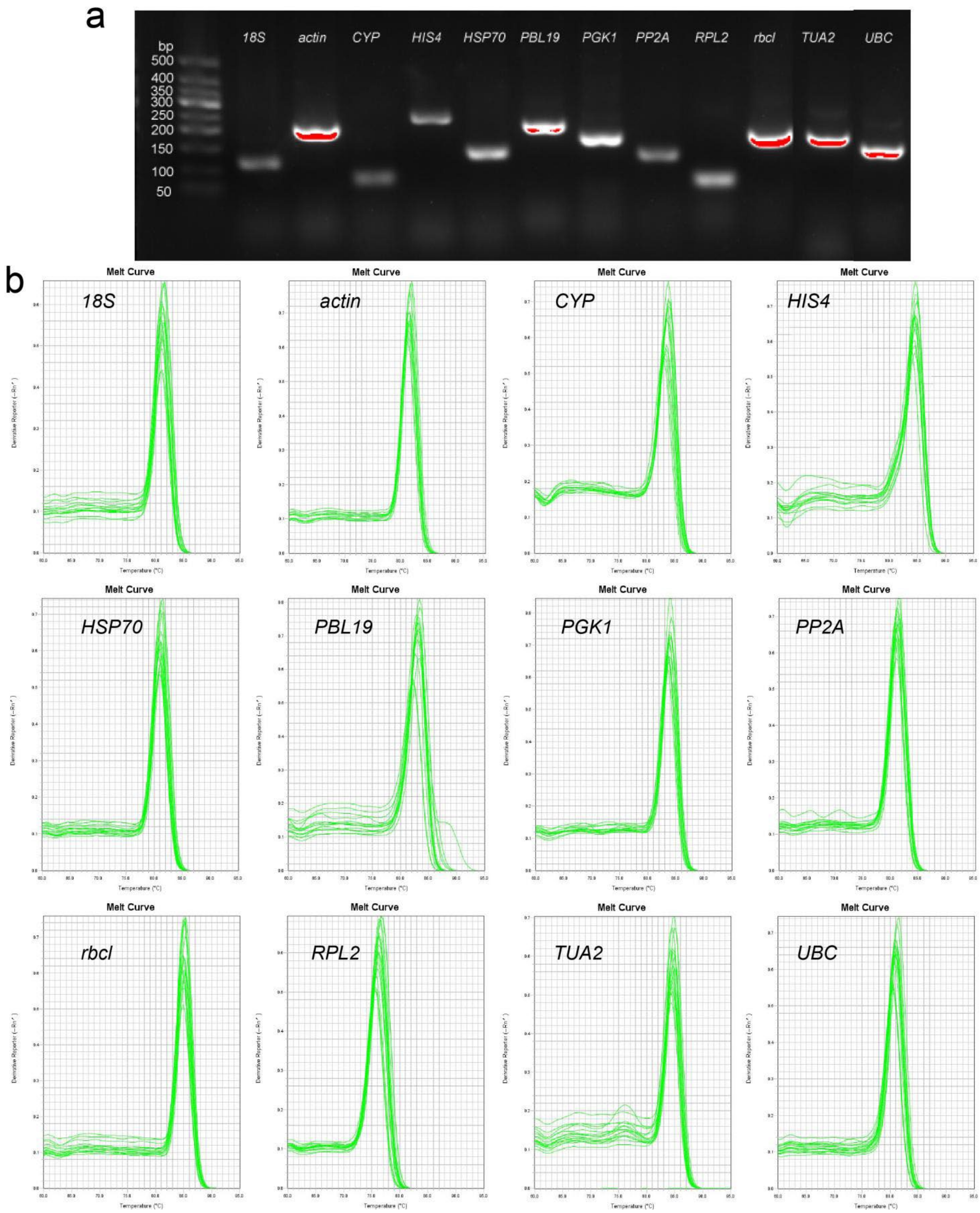

Figure S1. Specificity of each candidate reference gene primer pair. (**a**) 2.5% agarose gel showing that all primer pairs exhibited specificity and that the sizes of the amplification products were consistent with the expected sizes. (**b**) Melting curves of the twelve candidate *C. fortunei* reference genes. *18S, 18S ribosomal RNA*; *CYP, cyclophilin*; *HIS4, histone H4*; *HSP70, heat shock protein 70*; *PBL19, serine/threonine-protein kinase*; *PGK1, phosphoglycerate kinase 1*; *PP2A, protein phosphatase 2A*; *rbcl, large subunit of ribulose-1,5-bisphosphate carboxylase/oxygenase*; *RPL2, 60S ribosomal protein L2*; *TUA2, tubulin alpha-2*; *UBC, ubiquitin-conjugating enzyme*.

Table S1. Statistics for genes with SD values ≥ 1 under different treatments/in different tissues.

Rank	Gene	Number
1	*TUA2*	7
2	*HIS4*	6
3	*PGK1*	4
4	*rbcl*	3
5	*RPL2*	3
6	*PP2A*	2
7	*HSP70*	2

HIS4, histone H4; HSP70, heat shock protein 70; PGK1, phosphoglycerate kinase 1; PP2A, protein phosphatase 2A; rbcl, large subunit of ribulose-1,5-bisphosphate carboxylase/oxygenase; RPL2, 60S ribosomal protein L2; TUA2, tubulin alpha-2.

Table S2. Statistics for the last three RGs in the stability rankings according to the two comprehensive evaluation methods.

Rank	Geometric mean		RefFinder	
	Gene	Number	Gene	Number
1	*TUA2*	12	*TUA2*	12
2	*PGK1*	9	*PGK1*	9
3	*HIS4*	8	*HIS4*	7
4	*rbcl*	4	*rbcl*	4
5	*RPL2*	1	*RPL2*	2
6	*PP2A*	1	*PP2A*	1
7			*HSP70*	1

HIS4, histone H4; HSP70, heat shock protein 70; PGK1, phosphoglycerate kinase 1; PP2A, protein phosphatase 2A; rbcl, large subunit of ribulose-1,5-bisphosphate carboxylase/oxygenase; RPL2, 60S ribosomal protein L2; TUA2, tubulin alpha-2.

Table S3. Top five genes ranked by the five tools.

Method	Rank	Cold	Heat	Drought	Salt	ABA	GA_3	MeJA	SA	Tissue	Abiotic	Hormone	Total
delta Ct	1	*actin*	*CYP*	*CYP*	*rbcl*	*actin*	*UBC*	*UBC*	*actin*	*actin*	*CYP*	*CYP*	*CYP*
	2	*rbcl*	*UBC*	*HSP70*	*18S*	*CYP*	*CYP*	*CYP*	*CYP*	*PBL*	*actin*	*actin*	*actin*
	3	*CYP*	*HSP70*	*actin*	*actin*	*UBC*	*PBL*	*actin*	*UBC*	*CYP*	*HSP70*	*UBC*	*UBC*
	4	*18S*	*PBL*	*UBC*	*HSP70*	*18S*	*actin*	*PBL*	*rbcl*	*HSP70*	*UBC*	*PBL*	*PBL*
	5	*UBC*	*actin*	*18S*	*CYP*	*rbcl*	*RPL2*	*PP2A*	*RPL2*	*UBC*	*PBL*	*RPL2*	*HSP70*
geNorm	1	*CYP*	*CYP*	*HSP70*	*18S*	*actin*	*CYP*	*18S*	*CYP*	*HSP70*	*actin*	*actin*	*actin*
		rbcl	*UBC*	*CYP*	*rbcl*	*CYP*	*UBC*	*UBC*	*UBC*	*CYP*	*UBC*	*UBC*	*UBC*
	3	*actin*	*PBL*	*actin*	*actin*	*UBC*	*PBL*	*actin*	*actin*	*PBL*	*CYP*	*CYP*	*CYP*
	4	*18S*	*HSP70*	*UBC*	*HSP70*	*rbcl*	*HSP70*	*CYP*	*RPL2*	*actin*	*18S*	*HSP70*	*HSP70*
	5	*UBC*	*actin*	*18S*	*CYP*	*18S*	*actin*	*PBL*	*rbcl*	*UBC*	*HSP70*	*RPL2*	*18S*
NormFinder	1	*rbcl*	*CYP*	*CYP*	*rbcl*	*actin*	*UBC*	*CYP*	*CYP*	*actin*	*CYP*	*UBC*	*CYP*
	2	*CYP*	*UBC*	*HSP70*	*18S*	*CYP*	*CYP*	*UBC*	*UBC*	*PBL*	*UBC*	*actin*	*UBC*
	3	*actin*	*HSP70*	*actin*	*actin*	*18S*	*PBL*	*actin*	*rbcl*	*HSP70*	*18S*	*CYP*	*actin*

	4	*18S*	*PBL*	*UBC*	*HSP70*	*UBC*	*18S*	*PBL*	*actin*	*CYP*	*HSP70*	*rbcl*	*HSP70*
	5	*UBC*	*PP2A*	*18S*	*CYP*	*rbcl*	*actin*	*PP2A*	*RPL2*	*PP2A*	*actin*	*HSP70*	*18S*
BestKeeper	1	*CYP*	*PP2A*	*actin*	*18S*	*rbcl*	*18S*	*UBC*	*UBC*	*18S*	*18S*	*rbcl*	*18S*
	2	*UBC*	*CYP*	*18S*	*rbcl*	*PP2A*	*CYP*	*18S*	*rbcl*	*UBC*	*UBC*	*UBC*	*UBC*
	3	*actin*	*18S*	*UBC*	*actin*	*UBC*	*rbcl*	*rbcl*	*CYP*	*actin*	*actin*	*18S*	*actin*
	4	*rbcl*	*HSP70*	*CYP*	*UBC*	*18S*	*RPL2*	*PP2A*	*actin*	*CYP*	*CYP*	*CYP*	*CYP*
	5	*18S*	*UBC*	*HSP70*	*CYP*	*CYP*	*UBC*	*CYP*	*RPL2*	*PBL*	*PP2A*	*actin*	*PP2A*
RefFinder	1	*CYP*	*CYP*	*CYP*	*rbcl*	*actin*	*UBC*	*UBC*	*CYP*	*actin*	*actin*	*CYP*	*CYP*
	2	*rbcl*	*UBC*	*HSP70*	*18S*	*CYP*	*CYP*	*CYP*	*UBC*	*CYP*	*CYP*	*UBC*	*actin*
	3	*actin*	*HSP70*	*actin*	*actin*	*rbcl*	*PBL*	*18S*	*actin*	*PBL*	*UBC*	*actin*	*UBC*
	4	*UBC*	*PP2A*	*UBC*	*HSP70*	*UBC*	*18S*	*actin*	*rbcl*	*HSP70*	*18S*	*rbcl*	*18S*
	5	*18S*	*PBL*	*18S*	*CYP*	*18S*	*actin*	*PP2A*	*RPL2*	*UBC*	*HSP70*	*PBL*	*PBL*
